# Monitoring the Setting Process of Cementitious Materials Using Guided Waves in Thin Rods

**DOI:** 10.3390/ma14030566

**Published:** 2021-01-25

**Authors:** Dongquan Wang, Guangyun Yu, Shukui Liu, Ping Sheng

**Affiliations:** 1 State Key Laboratory for Geomechanics and Deep Underground Engineering, China University of Mining and Technology, Xuzhou 221116, China; wdq8785@cumt.edu.cn (D.W.); skliu@cumt.edu.cn (S.L.); 2 School of Mechanics and Civil Engineering, China University of Mining and Technology, Xuzhou 221116, China; shengping@cumt.edu.cn

**Keywords:** guided waves, setting time, mortar and concrete, early age

## Abstract

Characterizing early-age properties is very important for the quality control and durability of cementitious materials. In this paper, an approach using embedded guided waves was adopted to monitor the changes in the mechanical proprieties of mortar and concrete during setting, and embedded thin rods with low-cost piezoelectric sensors mounted on top were used for guide wave monitoring. Through continuous attenuation monitoring of the guided waves, the evolution of mortar and concrete properties was characterized. Four different kinds of metallic rods were tested at the same time to find out the optimal setup. Meanwhile, shear wave velocities of the mortar and concrete samples were monitored and correlated to the attenuation, and setting time tests were also performed on these samples. Experimental results demonstrate that the proposed approach could monitor the evolution of the setting of cementitious materials quantitatively, and time of the initial setting could be determined by this technique as well. In addition, it is found that the attenuations of fundamental longitudinal guided wave mode are almost the same in concrete samples and mortar samples sieved from concrete, indicating that this technique is able to eliminate the effects of coarse aggregates, which makes it of great potential for in-situ monitoring of early age concrete.

## 1. Introduction

In reinforced concrete structures, the workability and durability of concrete are critical for the structures to meet desired structural performance [1,2,3]. Furthermore, the evaluation of properties, such as the setting time of fresh concrete, is critical for assuring quality and reducing construction time. Conventionally, the penetration test that measures the shear resistance of the cementitious material was widely used to determine setting times [4]. In addition, research also showed the possibility of using ultrasonic body waves (longitudinal and shear waves) to characterize the early age properties of cementitious material [5,6,7,8,9,10,11,12,13,14].

Lots of research has been conducted to correlate the body wave velocities and material properties. For instance, many efforts were made, aiming to find the relationship between primary wave velocity and setting times [9,11,15,16,17,18,19]. Dumoulin et al. [19] used embedded piezoelectric patches as smart aggregates, to monitor the primary wave velocity evolution during the setting and hardening phases of concrete, and it was found that it was hard to extract the primary wave velocity with a good accuracy at very early ages. Besides, water in the cementitious materials leads to a high primary wave velocity at the early age, which will shield the velocity originating from the solid portion of the material during setting [20]. Moreover, recent research showed that the primary wave velocity in cementitious material is affected by the presence of air void [11,21]. Research by Zhu et al. showed that the presence of air void has limited influence on the shear wave propagation, and the shear wave velocities are almost consistent at the initial setting [5,21,22], and this consistency was verified on cement pastes and mortar samples. Liu et al. [5] used an embedded bender element to monitor the shear wave velocity change during the setting of mortar and concrete samples, and a strong correlation between the shear wave velocity and penetration resistance was found. Carette determined the setting time of mortars containing two types of fly ashes based on the combined monitoring of P and shear waves, and it was found that shear wave velocity and dynamic elastic properties are the most accurate indicators of the setting process [23]. However, when it comes to the complexity of the implementation of the measurement system, the techniques above seem not that attractive.

Ultrasonic guided waves are elastic waves propagating in a plate or rod. Due to its long propagation range, the guided wave is frequently used in material characterization and damage detection in plates and pipes [24,25,26,27,28]. For instance, Zima and Kedra [27] carried out a series of numerical studies to find out the effect of a concrete mesostructure on lamb wave propagation in concrete plates, and it was found that the displacements associated with wave motion are affected by the mesostructure of the concrete plates. Lee et al. [28] formed an embedded guided wave sensor system by placing two pairs of collocated identical piezoelectric patches on a steel plate with uniform thickness. The system was then used for the hardening process monitoring of the ultra-high performance concrete. It was found that two features of the guided wave, namely the amplitude attenuation and the time-of-flight, are more suitable to monitor the behavior of the ultra-high performance concrete. When a rod is embedded in another medium, part of the guided wave energy will propagate through the interface, and leaky waves are excited in the surrounding medium. The attenuation caused by the leakage depends on the properties of the surrounding materials [29]. Thus, it is possible to evaluate the properties of the surrounding materials by monitoring the attenuation change. The through-transmission method was adopted by Sharma and Mukherjee [30,31] to measure guided waves in a rebar, and the signal amplitude was used to correlate with P wave velocity and compressive strength in concrete. Ervin et al. [32] used this method to monitor corrosion of rebar embedded in mortar. However, this method is not applicable when only one side could be accessed. Vogt et al. [29] investigated the scattering of ultrasonic guided waves at a point where a free cylindrical waveguide enters an embedding material, and the lowest-order longitudinal mode was recommended for embedded guided wave monitoring. As an example, Vogt et al. [33] used a steel wire for guide wave propagation, in a reflected manner, to monitor the curing process of epoxy resins, and the reflected guided wave signals were analyzed using both the reflection coefficient and attenuation method. It was found that both methods are sensitive to the shear properties at low frequencies. Sun and Zhu [34] improved the dispersion calculation of the embedded rebar and used the embedded rebar for guide wave propagation to monitor the early age properties of cement and mortar samples, and in this study the guided wave was generated using an electric coil and received by a commercial ultrasonic transducer. However, to the authors’ knowledge, only a steel rebar or wire with a fixed diameter was investigated in the studies above; it would be interesting to make comparisons between rods of different materials and diameters.

In this paper, a series of experimental tests on mortar and concrete samples with various mix designs were performed. Four different kinds of metallic rod were tested simultaneously to find out the optimal guided wave setup to monitor the setting process. Meanwhile, shear wave velocities of the mortar and concrete were monitored, and the time of setting was also measured from these samples. The relationship between these measurements was also discussed.

## 2. Materials and Methods

### 2.1. Materials

Two types of samples, namely mortar and concrete samples, were mixed, and ordinary Portland cement was used in both types of samples. The fine aggregate used in the two types of samples was river sand, and the relative density of this sand was 2.62. The coarse aggregate used in concrete samples was a dolomitic limestone with a relative density of 2.65. As shown in Table 1, the mortar samples were designed to span three water to cement ratios (w/c = 0.4, 0.45 and 0.5), and the w/c of the concrete sample was fixed to 0.5 to figure out the influence of coarse aggregate on the guided wave technique.

### 2.2. Ultrasonic Test Setup 

**Figure 1:**
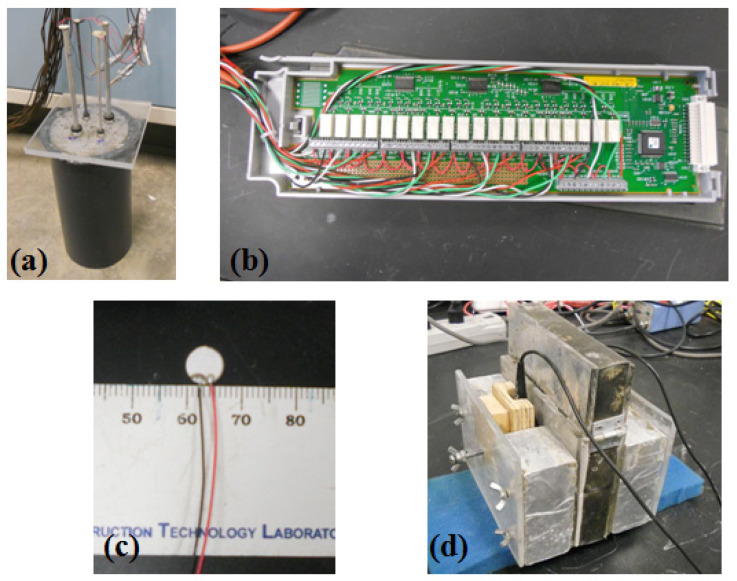
Ultrasonic Test setups: (**a**) guided wave testing; (**b**) 34903A switching card; (**c**) 7 mm diameter piezo disc and (**d**) shear wave measurement.

To find out the optimal material with appropriate mechanical properties and dimensions, four different metallic rods (namely steel rods with a diameter of 3.17 mm and 6.35 mm, and aluminum rods with a diameter of 3.17 mm and 6.35 mm) were embedded into the samples during the tests (see Figure 1a). The length of each rod was 304.8 mm, and the embedded length was 152.4 mm. A Plexiglas plate with four holes was used to keep the rods in place and to reduce the moisture evaporation as well. Low cost small piezo discs (SMD07T02S412, manufactured by STEINER & MARTINS, Inc., Miami, FL. USA) (see Figure 1a,c) bonded on top of the rods were used to excite the rods in the axial direction and to receive the reflected signals. In order to get the signals from the four rods alternately, an Agilent multiplexer 34970A with the plug-in 34903A card (Agilent, Santa Clara, CA, USA) (see Figure 1b) was used to connect these four piezo discs to a pulser-receiver (Olympus 5077PR, Tokyo, Japan) and a digitizer (NI-PXI 5133, Austin, TX, USA). The 34903A card is simply a set of 20 independent single-pole, double-throw reed relays, and each of the 20 relays could be independently controlled. In this study, only four relays were used and closed alternately to get the signals from the four rods. In order to reduce the noise ratio, a total of 200 signals obtained from each rod were averaged separately and then saved each time.

Meanwhile, shear wave testing was carried out using two shear wave transducers (Olympus V151, Olympus, Tokyo, Japan) in the same batch of samples independently. The setup for shear wave testing was the same as the one used in the author’s previous work [5], as shown in Figure 1d, the mold was formed by two plastic plates on the sides and a rubber container in between. A soft foam layer was installed between the internal container and external plastic plate, aiming to reduce the direct transmitted ultrasonic waves. Two shear wave transducers were put on each side of the mold. In this setup, the sample thickness was about 27 mm, and the actual thicknesses will be tested after shear wave testing. During the shear wave testing, one transducer served as an actuator, and the other was used as a receiver. Both transducers were connected to an Olympus 5077PR pulser-receiver (Olympus, Tokyo, Japan), with a gain of 40 dB. A 200 V square wave pulse sent from the pulser-receiver was used to drive the actuating transducer. The receiving signals were then sampled at a 10 MHz sampling rate by a NI-USB 5133 digitizer (National Instruments, Austin, TX, USA), which was also used to transfer digitized signals to the connected computer. Furthermore, 200 signals were first obtained and then averaged, as used in the guided wave testing setup, to reduce the noise ratio. Both shear wave velocity tests and guided wave tests were carried out with a 5 min interval until the final setting of the samples.

### 2.3. Penetrometer Tests

In order to determine the initial and final setting of the samples, penetration resistance tests were carried out on the same batch of mortar and concrete samples, according to ASTM C403 [4]. For concrete samples, sieving is needed, and the sieved mortar was obtained by wet-sieving the selected portion of concrete through a 4.75-mm sieve and onto a non-absorptive surface. In the penetration resistance tests, mortar samples were penetrated using standard needles, and the resistance value was obtained at regular time intervals. Then a plot of penetration resistance versus time was used to determine the times of initial and final setting. The initial and final times of setting correspond to penetration resistance values of 3.5 MPa and 27.6 MPa, respectively. The setting times of all the mixtures are also listed in Table 1.

## 3. Results and Discussions

### 3.1. Shear Wave Velocity Testing

It has been shown that the propagation of the shear wave is less affected by the presence of air void in cementitious materials. Thus, only the shear wave velocity is obtained and analyzed in this study. It was noticed that it is hard to pick the first arrival of shear waves from a single piece of signal in the time domain. Thus, B scan images were formed by a stack of signals in the time domain together and used to capture the shear wave arrival. The shear wave arrival time was first obtained using a MATLAB (R2019b, The MathWorks, Inc., Natick, MA, USA) input function to manually pick arrival points at different ages along the arrival time trend. Then, the shear wave velocity was calculated by dividing the thickness of the mortar sample, which was 27 mm in this study, over the travel time of the wave through the sample. Note that a test system time of 4 μs was subtracted to obtain the actual travel time.

**Figure 2:**
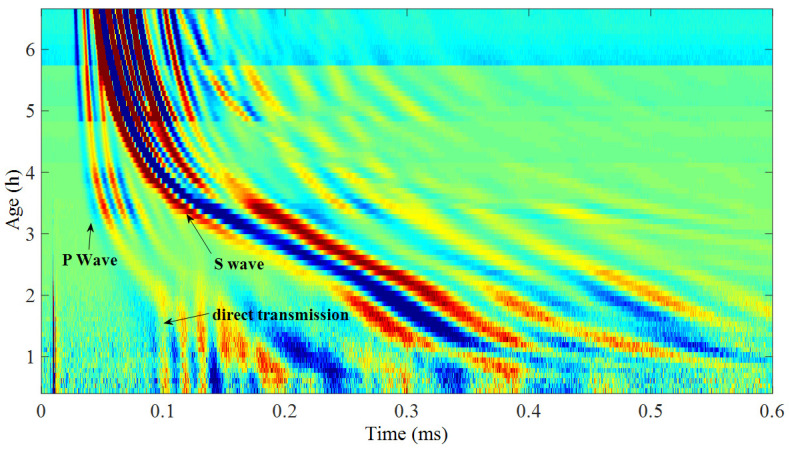
B scan image formed from signals getting from a mortar sample (w/c = 0.40).

Figure 2 shows a B scan image of wave propagation evolvement during setting in a mortar sample (w/c = 0.40), the x-axis and y-axis in the figure representing the time of signals and the age of mortar, respectively. It was noticed that at the first 2.5 h of setting, direct transmission waves transmit along the rubber mold are stronger than the waves propagating directly through the sample. However, by using B scan images, the first arrivals at a very early age can still be obtained.

**Figure 3:**
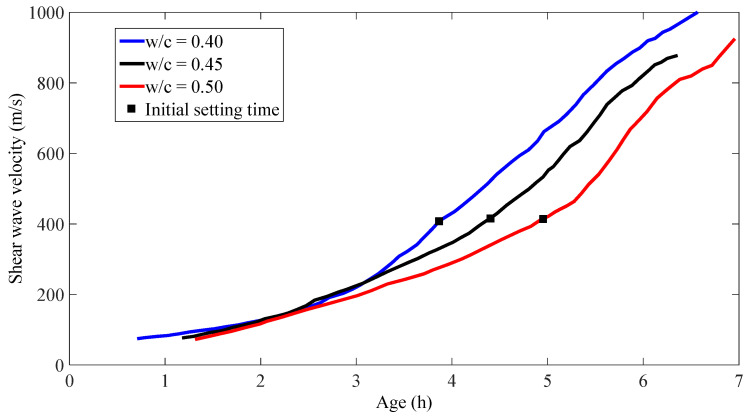
Shear wave velocities in different w/c mortar samples.

Shear wave velocities obtained from different w/c mortar samples are shown in Figure 3, and the times of the initial setting are also marked on the corresponding curves. It is clearly seen that as w/c increases, a much longer initial setting time is taken, and it was also noticed that even though the w/c are different, the shear wave velocities are very consistent (around 410 m/s) at initial setting times. This trend agrees very well with previous research on cement paste [22].

### 3.2. Guided Wave Testing

**Figure 4:**
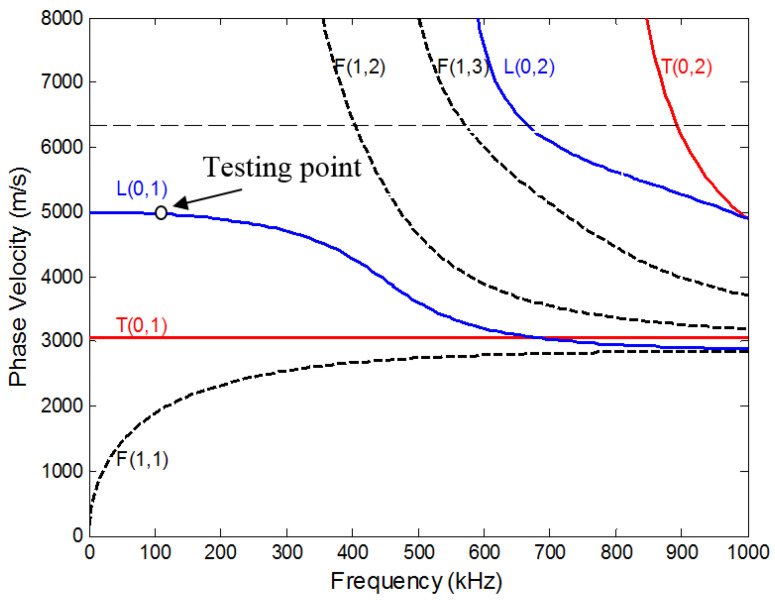
Phase velocity dispersion curves for an aluminum rod of 6.35 mm diameter.

Many guided wave modes exist in a cylindrical rod, and each mode travels at a different velocity. These wave modes are often categorized into three different types: longitudinal (L), torsional (T) and flexural (F). Figure 4 shows the dispersion curves of the phase velocity for the first few modes in the 6.35 mm aluminum rod. The fundamental L (0, 1) mode was identified as most suitable for this study for several reasons. First of all, at the testing point (see Figure 4), there is mainly axial surface displacement and little radial displacement in the rod, which makes the attenuation very sensitive to the shear wave velocity of the surrounding mortar or concrete. Furthermore, most importantly, the L (0, 1) mode could be easily excited by the piezo discs adopted in this study and is non-dispersive at low frequency. The attenuation caused by the surrounding mortar or concrete could be calculated from:

(1) $$\alpha =-\frac{1}{D}\ln f_{0}(\frac{A_{\mathrm{emb}}}{A_{0}})$$

where D is the embedded depth, A_0_ is the signal amplitude before the rod was embedded, and A_emb_ is the signal amplitude after the rod was embedded.

**Figure 5:**
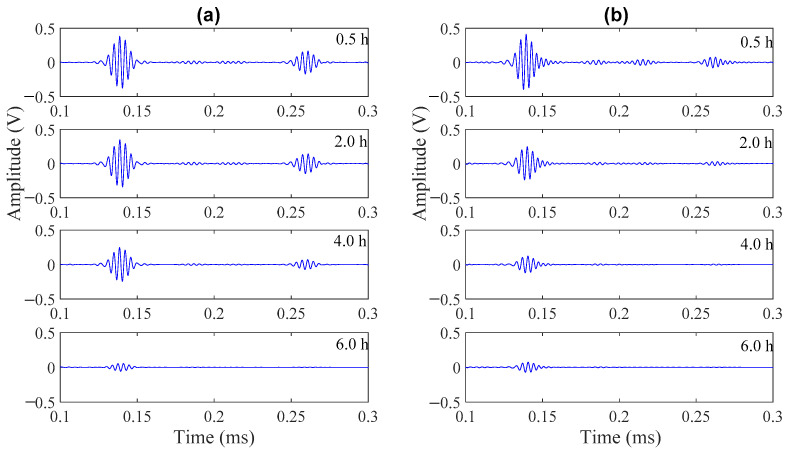
Waveforms at 0.5, 2.0, 4.0, and 6.0 h for a mortar sample with w/c = 0.40 using: (**a**) a steel rod of 3.17 mm diameter; (**b**) an aluminum rod of 3.17 mm diameter.

Figure 5 shows two sets of representative waveforms for a mortar sample with w/c of 0.40 using different rods, and only the first two reflection echoes were shown. As shown in Figure 5, amplitudes of the signals getting from the steel rod of 3.17 mm diameter decrease very slowly in the first 4 h. In contrast, the amplitudes of the signals getting from the aluminum rod of 3.17 mm diameter decrease much faster in the first 4 h. This is probably due to the reason that acoustic impedance mismatch between early age mortar and aluminum is much smaller than the mismatch between early age mortar and steel. Thus, more energy is leaked to the surrounding mortar sample in the aluminum rod. This also implies that the aluminum rod is probably more sensitive and suitable to monitor the evolution of early age properties of cementitious samples.

**Figure 6:**
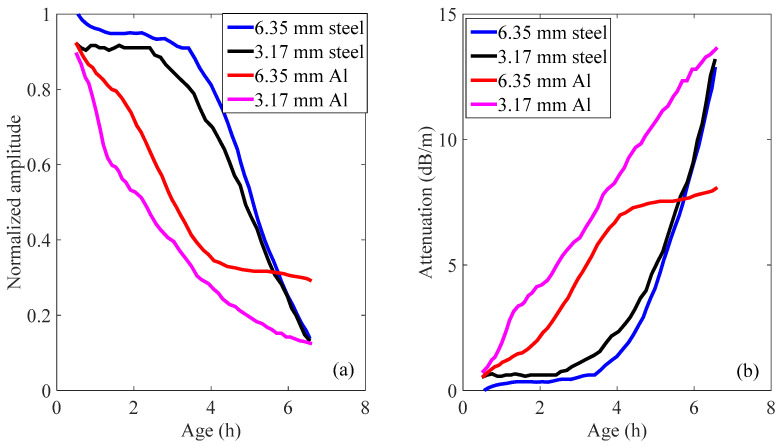
Four different rods in mortar sample with w/c = 0.40. (**a**) normalized wave amplitude and (**b**) attenuation.

The amplitude of the reflected guided waves was then normalized to the amplitudes of the waves obtained when the rods were not embedded. Figure 6a shows the normalized peak amplitudes getting from four different rods as a function of mortar age. It was noticed that during the first 3 h, the peak amplitudes of both the 6.35 and 3.17 mm steel rods decrease very slowly. As a result, the guided wave attenuation in these two rods does not increase significantly in the first 3 h (see Figure 6b).

**Figure 7:**
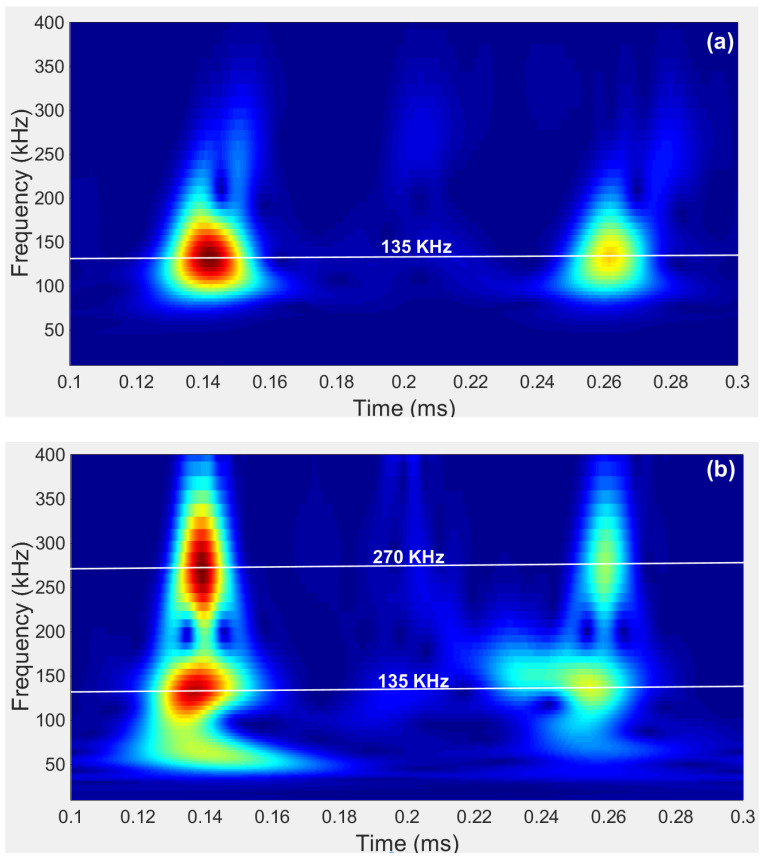
The wavelet of signals obtained from mortar sample with w/c = 0.40 at the age of 0.5 h. (**a**) steel rod with a diameter of 6.35 mm and (**b**) steel rod with a diameter of 3.17 mm.

Another phenomenon noticed is that the attenuation trends in both steel rods are almost the same, especially after 5 h of setting. The reason was further investigated, and wavelet analysis was adopted to see the time-frequency domain difference between the signals obtained from the 6.35 mm and 3.17 mm diameter steel rods, respectively. As seen in Figure 7a, the central frequency of the reflected echoes obtained from the 6.35 mm diameter steel rod is 135 kHz, corresponding to a frequency-radius product of 428.63 kHz-mm. Sun [34] has proposed an improved approach to calculate the group velocity dispersion curve of rebar embedded into concrete, based on the PCDISP package [35]. By using Sun’s approach, the theoretical group velocity in steel rod with a frequency-radius product of 428.63 kHz-mm was found to be 4989 m/s. While the group velocity calculated using the experimental data in this study was 2 × 304.8 mm/(0.2623–0.1403) ms = 4997 m/s, this agrees well with the theoretical group velocity.

In the reflected echoes obtained from the 3.17 mm diameter steel rod, two dominant central frequencies, 135 kHz and 270 kHz could be observed. Note that the frequency-radius product of the 270 kHz signal is 428.63 kHz-mm in the 3.17 mm rod, and this product is the same as the one obtained from the 6.35 mm rod. As the hardening process develops, the 428.63 kHz-mm product will gradually become the dominant one in the 3.17 mm rod. Thus, the attenuation trends in both the 6.35 mm and 3.17 mm diameter steel rods are almost the same after 5 h of setting.

As seen in Figure 6, both the 6.35 mm and 3.17 mm aluminum rods show a rapid decrease in peak amplitudes in the first 4 h. Note that from around four hours, the decrease rate of the peak amplitude of the 6.35 mm aluminum rod becomes very slow. While the decrease rate of the peak amplitude of the 3.17 mm aluminum rod remains pretty high, this continuous rapid change makes the 3.17 mm aluminum rod the best choice within the four rods for monitoring the setting of cementitious materials. For simplicity, only the results obtained from the 3.17 mm aluminum rod will be shown in the following sections.

Since there is mainly axial surface displacement and little radial displacement under the low- frequency L (0,1) mode [36], shear leakage will control the attenuation caused by the embedded materials. Therefore, this mode is particularly sensitive to the shear properties of the embedded materials. Note that the shear wave velocity was obtained during the test as well, it would be very interesting to correlate it with the guided wave attenuation.

**Figure 8:**
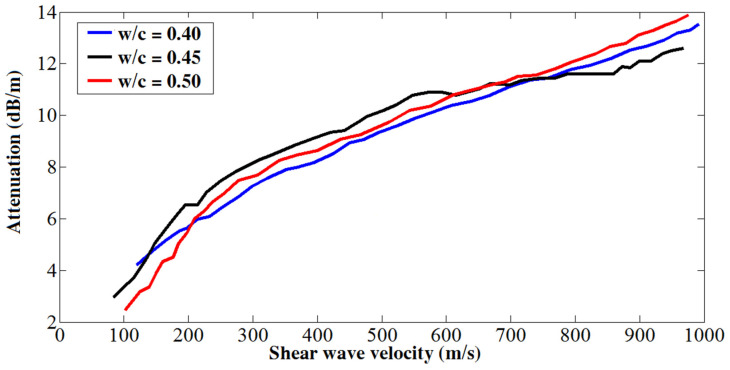
Correlation between shear wave velocity and attenuation.

Figure 8 shows the correlation formed by shear wave velocity versus the guided wave attenuation. As seen in the figure, despite some variations in the very early age, shear wave velocity still correlates strongly with the guided wave attenuation. Specially, after the very early age, there is an approximately linear relationship between these two measurements. Similar relationships, obtained by using steel rod for guide wave propagation, were also found in the hardening process monitoring of epoxy resins [33] and cement pastes [34].

**Figure 9:**
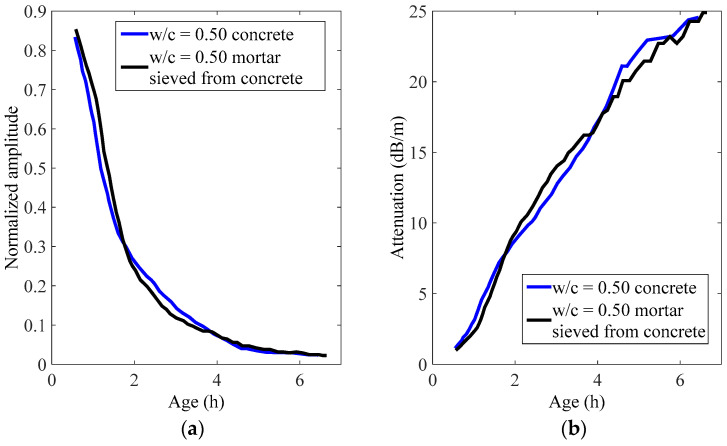
Guided waves in concrete and mortar sieved from concrete: (**a**) normalized amplitude and (**b**) attenuation.

To find out the influence of coarse aggregates on this technique, a w/c = 0.50 concrete sample was tested in this study. The normalized amplitude and attenuation change during hydration of a concrete sample, and the mortar sample sieved from concrete are shown in Figure 9. As shown in the figure, the developing trends in both materials are almost identical. This is probably because the rod is very thin compared to the size of coarse aggregates, and the rod is still mainly surrounded by mortar when embedded in the concrete sample. This implies that this technique is able to eliminate the effects of coarse aggregates, which makes it of great potential to be applied to the fresh concrete in the field after a standard setup is established. Another thing that needs to be noted is that an increasing practice in concrete engineering is the use of additives to reduce the water dosage, and the rheological properties of concrete samples using additives may be quite different. Thus, extensive attention should be paid when applying this approach to concrete samples using additives.

**Figure 10:**
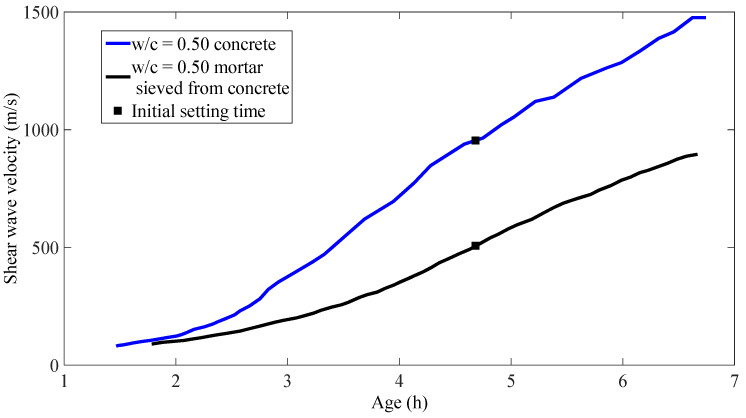
Shear wave velocities in concrete and mortar sieved from concrete.

In order to find out whether coarse aggregates affect the development and the evolution of shear waves in concrete, shear wave velocity change during the setting process is also shown here (see Figure 10). As seen in the figure, the shear wave velocity in concrete is around 860 m/s at the initial setting time, and it is about 450 m/s higher than the shear wave velocity in mortar sieved from the same batch of concrete. This is understandable, because when taking shear wave velocity measurement on concrete samples, the shear wave has to propagate through the coarse aggregates, whose mechanical properties are very different from the surrounding mortar. In other words, the effects of coarse aggregates could not be ignored when taking shear wave velocity measurements.

## 4. Conclusions

In this study, an embedded guided wave technique has been adopted to monitor the setting process of cementitious samples. Specially, four different kinds of rods have been tested simultaneously to find out the optimal guided wave setup. Shear wave velocities of the mortar and concrete have been monitored and the time of setting has also been measured on these samples, and the relationship between these measurements has been discussed. The following conclusions could be drawn from this study.

Within the four rods tested in this study, the aluminum rod with a diameter of 3.17 mm performs the best. It could be adopted to characterize the whole setting process of cementitious samples with standard water to cement ratios.

By using an aluminum rod in the fundamental mode, a strong correlation between the attenuation and the shear wave velocity has been shown in mortar samples. This trend agrees well with the previous embedded guide wave monitoring studies, which used a steel rod to guide wave propagation.

This technique worked equally well in concrete samples and mortar samples sieved from the same batch of concrete. This implies that this technique is able to eliminate the effects of coarse aggregates, and is of great potential for field setting monitoring of cementitious materials with standard water to cement ratios.

## Figures and Tables

**Table 1:** Mixture designs and setting times of mortar and concrete samples.

*w*/*c*	Sample Type	Coarse Aggregate Volume (%)	Fine Aggregate Volume (%)	Initial Setting Time (min)	Final Setting Time (min)
0.40	mortar	–	51.1	232	342
0.45	mortar	–	51.1	264	371
0.50	mortar	–	51.1	297	399
0.50	concrete	43.4	28.9	281	384

–not applicable.

## Data Availability

Data available on request due to their size properties. The data presented in this study are available on request from the corresponding author.
